# Circulating natural killer cells and their association with breast cancer and its clinico-pathological characteristics

**DOI:** 10.3332/ecancer.2023.1567

**Published:** 2023-07-03

**Authors:** Homian N’da Marcelin, Romuald S Dasse, Richard O Yeboah, Agnès D Tariam, Arsène G Z Kagambega, Akandji M Oseni, Y K K Kouassi, Michel A Bilé, Moctar Toure, Monica Thakar, Innocent Adoubi, Darya Kizub

**Affiliations:** 1 Department of Oncology, Centre Hospitalier Universitaire de Treichville, 7XVV+5P4, Abidjan, Côte d’Ivoire; 2 Department of Immunology, Centre Hospitalier Universitaire de Cocody, BP V 3, Abidjan, Côte d’Ivoire; 3 The Fred Hutchinson Cancer Center, Seattle, WA 98109, USA; 4 University of Texas MD Anderson Cancer Center, Houston, TX 77030, USA

**Keywords:** natural killer (NK) cells, breast cancer, treatment

## Abstract

**Purpose:**

Natural killer (NK) cells play a critical role in cancer immunosurveillance and hold promise as both therapies and prognostic markers in advanced disease. We explore factors that may influence NK cell concentration in the peripheral blood of women with breast cancer in Côte d’Ivoire compared to healthy controls and implications for future research in our context.

**Methods:**

In this cross-sectional case-control study, blood samples were taken from 30 women diagnosed with breast cancer within 6 months of diagnosis and fifteen healthy women at University Teaching Hospital [Centre Hospitalier Universitaire (CHU)] Treichville in Abidjan, Côte d’Ivoire, from March to September 2018. The blood draw could take place at any time following diagnosis and through treatment. Demographic and clinical data were collected. NK cells were isolated, stained, analysed and counted using the flow cytometer at the Department of Immunology at CHU of Cocody. All p-values were two-sided.

**Results:**

Mean age among 30 women with breast cancer was 49 years old compared to 45 years old for 15 controls (*p* = 0.41). Among 30 women with breast cancer, 4 (13.3%) had Stage 2 disease, 14 (46.7 %) at Stage 3, and 12 (40%) at Stage 4. Fourteen (46.7%) had breast cancer that was hormone receptor-positive (HR+) HER2-negative, 10 (33.3%) had triple-negative cancer, three (10.0%) had HR+HER2+ disease, and three (10.0%) HR-HER2+ cancer. NK cell concentration was not associated with cancer diagnosis, age, cancer stage, subtype, or type of treatment patients received (*p* > 0.05).

**Conclusion:**

Although we did not find an association between NK cell concentration, cancer characteristics or treatment, our results be limited by the small sample size and timing of blood draw. Our next steps include a larger study to explore circulating NK cells prior to any treatment and NK cell infiltration within breast cancer tumour and correlating this with response to treatment and prognosis.

## Background

Breast cancer is the most common cancer in women and the second most common cancer worldwide. Its burden is quickly rising in low- and low-middle-income countries [1, 2], where patients suffer from a high mortality rate reaching up to 80% in Sub-Saharan Africa [3]. This is due to more aggressive cancer subtypes, delayed presentation and the lack of adequate screening and treatment. Studies in African countries suggest low knowledge of breast cancer tending to impact on attitudes to uptake of screening and timely presentation when symptoms develop, resulting in late diagnosis [4, 5] leading to poor survival [6]. Invasive ductal carcinoma is the most common presentation, with younger age at onset and increased incidence of HER2/neu positive and triple negative and basal-like breast cancers compared to Europe and North America [7, 8].

The prognosis and classification of breast cancer are dependent on the tumour burden which takes into account tumour size, lymph node involvement, and distal metastasis [Tumour, Node, Metastasis (TNM)] [9]. A combination of surgery, radiotherapy, chemotherapy and targeted therapies can result in a cure or prolonged survival. Chemotherapy prevents the tumour from growing by targeting rapidly dividing cells. However, it affects both tumour cells and normal cells, often resulting in significant side-effects. Targeted therapies impact tumour growth and ability to metastasise by inhibiting specific cell-surface receptors or intracellular proteins in the downstream signalling pathways [10, 11]. Through a combination of these treatments can be effective, some breast cancers are diagnosed too late to be cured by surgery or recur after resection and eventually develop resistance to existing treatment.

Breast cancer is the most prevalent cancer in Côte d’Ivoire and accounts for 21.7% of all cancers and 34.9% of all cancers in women, with approximately 2,659 new cases, 4,960 5-year prevalence, and 1,317 deaths in 2018 [2]. A study of 350 women who were diagnosed with breast cancer 2008–2011 in Côte d’Ivoire found that 79% presented 10 months or later after noticing symptoms. The median age was 42 (range 18–81) and 45% had five or more children. Immunohistochemistry for HER2 status was done in 6%. Thirty-three (9.4%) presented with Stage IIB, 140 (40.0%) Stage IIIA, 127 (36.3%) with Stage IIIB, and 50 (14.3%) with Stage IV [12].

Though early detection of down-stage breast cancer is integral to improving outcomes, new approaches to the treatment of women with late-stage disease are urgently needed. Development of basic science and clinical research capacity in Côte d’Ivoire to develop and test novel treatments in the local context will be invaluable to helping improve survival and quality of life.

In order to grow and multiply, breast cancer cells have to evade the patients’ natural immune response [13]. The natural killer (NK) cell, a lymphocyte that is part of both the innate and the adaptive immune system, has garnered extensive interest as a potential avenue of enhancing the immune system response against both breast cancer and other solid and liquid malignancies [14, 15].

NK cells play a key role in cancer cell detection and destruction through the activation of proinflammatory pathways [16, 17], but have less activity in patients with breast cancer [18, 19] as a result of the hypoxic tumour microenvironment decreases NK cell activation and allows cancer cells to escape [19–21]. Survival in patients with breast cancer correlates with increased NK cell expression of activating markers; NK cell dysfunction and inhibitory surface cell protein markers correlate with cancer progression [22, 20].

Current clinical trials are exploring the potential of *ex-vivo* expanded and activated autologous NK cells where the inhibitory inactivated phenotype is reversed, autologous genetically modified NK cells, haploidentical hematopoietic stem cell transplant combined with donor NK cell infusion and targeted immunotherapy to activate the inhibitory NK cell phenotype to treat both solid and hematologic malignancies [14–24].

The main purpose of this study was to explore the profile of circulating NK cells in women diagnosed with breast cancer in Côte d’Ivoire, as well describing breast cancer characteristics and treatment received. The study objectives included characterising the relationship between NK cell concentration in peripheral blood and breast cancer stage, immunohistochemical type, and the type of treatment received. We also compared the influence of demographic characteristics on circulating NK cell concentration in women with breast cancer and healthy controls.

## Material and methods

### Study population and sample collection

This is a cross-sectional study comparing women with and without breast cancer. Samples of peripheral blood were taken from a sequential sample of 30 women diagnosed with breast cancer and from 15 women without breast cancer from March to September 2018 at the University Teaching Hospital (CHU) of Treichville, Côte d’Ivoire. Inclusion criteria for women with breast cancer were the following: age >18 years, female sex, diagnosis of breast cancer, receiving treatment at CHU Treichville, providing written informed consent for participation in the study and blood draw. Patients with another type of cancer were excluded. Inclusion criteria for women without breast cancer were the following: age >18 years, female sex, no history of cancer, no reported breast symptoms, no relationship to cases, residing in Treichville or Abidjan, providing written informed consent for study participation and blood draw. The study was offered to a sequential sample of patients at CHU Treichville; healthy controls were recruited by CHU Treichille staff through word-of-mouth. Participants’ demographic and clinical information, including information about breast cancer subtype and treatment received, was collected from hospital records. The Ki-67 was not tested due to the lack of reagent. Our patients received at least two of the following types of treatment: surgery, chemotherapy, hormonotherapy and targeted therapies (trastuzumab, bevacizumab). Peripheral blood samples were obtained at any time of any type of treatment from patients who gave a written informed consent. The interval time from the diagnosis to collection of blood sample was 6 months. The study protocol was approved by the Ethics Committee of the Ministry of Public Health and Hygiene.

### Peripheral blood mononuclear cells (PBMCs) isolation

Blood samples were collected from female patients with breast cancer and from female study participants without breast cancer in an anticoagulant citrate dextrose (ACD) Solution Becton Diskinson S.A Vacutainer. PBMCs were collected using a density gradient medium, Ficoll-isopaque Plus (GE Healthcare Life Sciences). The buffy coat, containing lymphocytes, was collected and cells were counted.

Cells were stained with Simultest CD3 FITC/CD16+56 PE. Then cells were washed with phosphate-buffered saline (PBS) solution, and analysed on Flow cytometer Model Facs calibur by Cell Quest Pro software. Cells were gated on CD56 + CD3– events (NK cells) and then analysed for the expression of other extracellular surface markers.

### Statistical analysis

Data entry was performed using Microsoft Excel^®^ 2013 software.

Clinicopathological variables were expressed as percentages or means with a SD and range. Student’s *T*-test was used to compare differences in means between two independent groups, while Pearson’s *R* test was used to test correlation between continuous variables assumed to be normally distributed (age, NK cell concentration). Univariate logistic regression was used to compare demographic and clinical characteristics between women with and without breast cancer. Participants with and without breast cancer were not matched based on demographic characteristics. The Mann–Whitney *U*-test was used to compare the distribution of NK cell concentration between two independent groups and the non-parametric 1-way ANOVA (Kruskal–Wallis test) for more than two groups given the small sample size. A two-sided *p*-value of < 0.05 was considered statistically significant. Statistical analyses were performed using IBM SPSS version 2.0 (French version).

## Results

### NK cells in women with and without breast cancer

**Figure 1.**
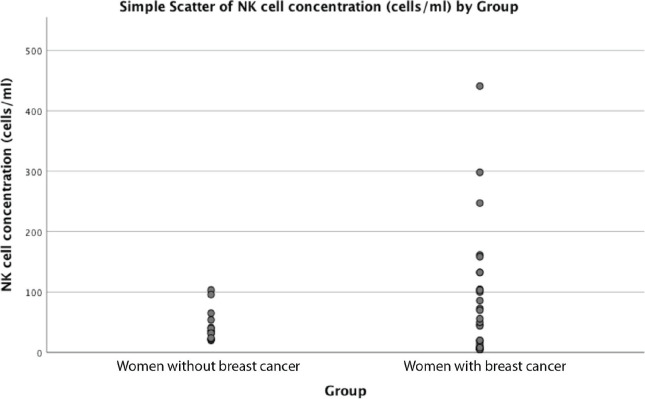
Relationship between NK cell concentration and breast cancer diagnosis.

Mean age was similar among the 30 women with breast cancer and 15 women without breast cancer, 49 versus 45 years old (*p* = 0.41). Mean NK concentration was higher in women with breast cancer compared to the group without breast cancer at 81 cell/mL versus 43 in controls, but this did not reach statistical significance (*p* = 0.054). (Table 1, Figure 1). NK cell concentration was not associated with age in either cases (*p* = 0.73) or controls (*p* = 0.93).

### Breast cancer stage and NK cells

Among 30 women with breast cancer, most (86%) had Stage III or de novo Stage IV disease. Mean NK cell concentration was 84 cell/mL for Stage II, 53 cell/mL for Stage III , and 112 cells/mL in stage IV (*p* = 0.35) (Table 2).

### Breast cancer immunohistochemical features and NK cells

**Figure 2.**
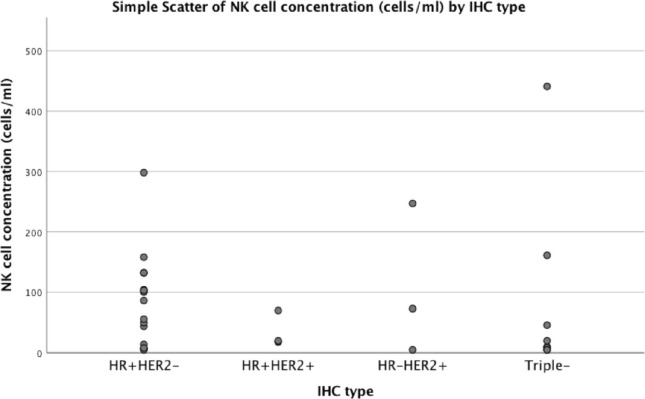
Relationship between NK cell concentration and breast cancer subtype.

Of the 30 women with breast cancer, nonspecific invasive carcinoma was observed in 29 (96.7%) of women with breast cancer and lobular carcinoma in one (3.3%). The most common breast cancer subtypes were hormone receptor-positive (HR+) HER2-negativ in 14 (46.7%) and triple-negative had triple-negative (HR-HER2−) in 10 (33.3%). The average NK cell concentration was 85 cells/mL in HR + HER2, 81 cells/mL in triple-negative, 66 cells/mL in HR + HER2+, 108 cells/mL HR-HER2+ (*p* = 0.78) (Table 3 and Figure 2).

### Breast cancer treatment and NK cells

Out of 30 women with breast cancer, 10 (33.3%) were treated with surgery, including nine with mastectomy and one with resection to negative margins.

Of 19 women with hormone-positive breast cancer, 10 (33.3%) received hormone therapy, including three receiving tamoxifen, three anastrozole, and one goserelin and tamoxifen.

Twenty-seven (90.0%) received chemotherapy, including all eight women with triple-negative breast cancer. Of 4 women with Stage 2 disease, one did not receive chemotherapy, whereas the other three received fluorouracil/epirubicin/cyclophosphamide (FEC) and docetaxel. Of the 14 women with Stage 3 disease, 6 received FEC 100 + docetaxel (4+4), 5 received AC (doxorubicin/cyclophosphamide), and 3 FEC 100 alone. Of the 12 women with Stage 4 disease, three received docetaxel + doxorubicin, two paclitaxel + carboplatin, whereas one each received AC, docetaxel + bevacizumab, doxorubicin + cisplatin, paclitaxel, paclitaxel + bevacizumab.

Five (16.7%) were treated with radiotherapy. Among women with stage 2 disease, two (50%) received radiotherapy. Among women with stage 3 disease, 3 of 14 (21.4%) received radiotherapy.

Five received targeted therapy (16.7%), including three receiving trastuzumab and two women with Stage IV disease receiving bevacizumab. Of four women with HER2+ disease, two received trastuzumab. Eight women (26.7%) received palliative care (Table 4).

There was no statistically significant correlation between NK cell concentration and the type of treatment women received (*p* > 0. 05) (Table 5).

## Discussion

Increased availability of breast cancer treatment and targeted therapies has improved patient outcomes. However, many women with breast cancer still succumb to this disease, especially in Côte D’Ivoire and other countries in Africa. This is due to a combination of late-stage disease at presentation and limited availability of treatment. In our study, 26 (86.3%) of the women presented with Stage III or IV disease, similar to other countries in the region [4, 5]. In our study, a larger percentage of women were of younger age and had triple-negative and HER2-positive disease, similar to other countries in the region [7, 8].

Of women with HR+ disease, over half received endocrine therapy. Of women with Stage III disease, about a third were able to receive radiation. Of women with HER2+ disease, half received trastuzumab. Over half of the women with Stage IV disease received palliative care. Insufficient access to treatment due to both cost and availability is similar to other countries in the region [25–28].

To improve outcomes for women with breast cancer in Côte D’Ivoire, an approach that would combine down-staging disease through early detection, improved access to existing treatment, and building local research capacity to facilitate the discovery and testing of novel treatments for cancers unresponsive to standard treatment is urgently needed.

Studying how the immune system could be activated to detect and kill breast cancer through NK cells may provide improved treatment options. Such discoveries may lead to the development of new drugs that can be used in combination with established therapies including chemotherapy, endocrine therapy, HER2-targeted treatments and immunotherapy [14–24].

Our study is the first in Côte D’Ivoire and in Sub-Saharan Africa to explore the relationship between NK cells and breast cancer. The goal was to explore the relationship between NK cells concentration and clinicopathological characteristics of patients with breast cancer, including the influence of treatment.

We did not find a relationship between NK cell concentration and age in either women with cancer or healthy controls. In a study done among men and women in Malaysia and Spain, NK cell number similarly either stayed the same or increased with age [29, 17].

As in studies conducted among patients in Spain and France that compared NK cell concentration in the peripheral blood of women with breast cancer and healthy controls, we did not find a statistically significant difference in the NK cell concentration these two groupds [30, 31]. There was a trend toward a higher average NK cell concentration in women with breast cancer. Interestingly, another study done in Brazil found a higher NK cell concentration in peripheral blood of women with cancer prior to chemotherapy, and a decrease in NK cell number following chemotherapy [32]. As in other studies, we found that NK cell concentration was not associated with tumour stage [30–32].

In our study, NK cell concentration did not vary by breast cancer immunohistochemical type. In another study, NK cell concentration similarly did not depend on oestrogen receptor status [33]. Another study found that patients with HER2 negative breast cancer had a higher NK cell concentration compared with those with an overexpression of the HER2 receptor [34].

It could be that we did not find a correlation between either NK cell concentration and breast cancer diagnosis or HER-2 status due to patients having received chemotherapy before the blood draw that resulted in fewer circulating NK cells.

Data from this preliminary study did not allow us to establish a relationship between the concentration of NK cells and treatment. Multiple prior studies evaluated the effect of different treatments on NK cell concentration in patients with breast cancer, though the timing of blood draw varied widely. NK cell concentration decreased significantly 2 weeks after chemotherapy in one study [35]. In another, a higher number of peripheral NK cells after neoadjuvant chemotherapy was associated with better clinical outcomes [36]. There was no difference in NK cell concentration between patients with breast cancer and healthy controls [37]. NK cell number did not change after chemotherapy, though they exhibited a less cytotoxic phenotype [37]. Endocrine therapy was associated with increased NK cell number in metastatic breast cancer [38]. NK cell concentration decreased in patients after radiation treatment but recovered 6 months later [39]. Decreasing NK cell counts after radiation were associated with worse outcomes [40].

Our results may have been limited by the small sample size and by the blood draw being done shortly after some patients have received chemotherapy. The women recruited may not have been representative of others with breast cancer in our country. However, since our results match those of other studies, we think our data is appropriately valid and provides interesting insights into NK cells and breast cancer and next steps for future research in our context.

Based on our findings, we are planning a larger research study that would standardise the timing of the blood draw at diagnosis and at a pre-determined point following treatment to examine the number of circulating NK cells and those within breast cancer tumours and correlate these with responses to treatment and prognosis. Reagents and antibodies to facilitate this research may be made available as part of a larger clinical trial of immunotherapy that will already examine tumour-infiltrating lymphocytes, including NK cells, to predict treatment response. The results may be of value in helping tailor treatment to risk of cancer recurrence or progression and enable us to simplify treatment regimens and decrease cost without compromising quality of care. Furthermore, efforts are already ongoing in Côte D’Ivoire to down-stage disease and improve access to treatment, though much more needs to be done.

Current public-private partnerships to improve cancer care in low- and low-middle income countries focus on clinician education and training, improving access to existing treatment modalities, and clinical guidelines and decision-making models to optimise outcomes the setting of limited resources [41–43]. There is less emphasis on research-related capacity building that would allow local clinicians to ask questions and execute projects relevant to their context. At the same time, discoveries made because of building research capacity in countries such as Cote d’Ivoire may help inform breast cancer treatment in settings where genetic variation may have an impact on treatment response. For example, though genetic variation does not appear to play a significant part in responses to chemotherapy and endocrine therapy in breast cancer, there is some evidence that it may have an impact on NK cell responses to cancer though the KIR receptor [44, 45].

Given the late stage of breast cancer at diagnosis and limited access to treatment in low-income and low-middle-income countries, actions should be taken by both governments and private companies in countries such as Côte d’Ivoire to make drugs available and affordable to not only improve the patients’ outcomes, but to use drugs in combination with NK cells to develop novel therapies [14–24].

Researchers at academic institutions and pharmaceutical companies in high-income countries involved in basic science or clinical research in cellular therapies and immunotherapy could lend their expertise to build local research capacity low-income and low-middle-income countries to explore and implement novel treatments for late-stage disease with the help of local oncologists, pathologists and epidemiologists.

## Conclusion

This is the first study of NK cell concentration in women with breast cancer compared to healthy controls in Cote d’Ivoire and Sub-Saharan Africa. Most women recruited for the study were diagnosed late in their disease and had insufficient access to existing treatment modalities. There was a trend toward higher NK cell concentration in women with breast cancer compared to healthy controls. NK cell concentration did not vary by cancer stage, subtype, or type of treatment. A large-scale study where the peripheral blood draw is done prior to any treatment is needed for the confirmation of this data in our context.

## Conflicts of interest

The authors declare no potential conflicts of interest.

## Presentation of results

These results have not been submitted elsewhere for publication. They were presented at the African Organization for Research in Cancer (AORTIC) Conference in 2021.

## Figures and Tables

**Table 1.** Participant characteristics and NK cell concentration.

Characteristic	Presence or absence of breast cancer	Mean	SD	Range	*p*-value
Age (years)	Participants with breast cancer	49	14	35–63	0.41
	Participants without breast cancer	45	17	26–65	
NK cell concentration (cells/mL)	Participants with breast cancer	81	101	5–441	0.054
	Participants without breast cancer	43	26	20–103	

**Table 2.** NK cell concentration and breast cancer stage.

Breast cancer stage at diagnosis	Number (%) of study participants (*n* = 30)	NK cell concentration (cells/mL)			
		Mean	SD	Range	*p*-value
II	4 (13.3)	84	54	8–132	0.35
III	14 (46.3)	53	108	7–158	
IV	12 (40.0)	112	185	5–298	

**Table 3.** NK cell concentration and breast cancer subtype.

Breast cancer subtype	Number (%) of study participants (*n* = 30)	NK cell concentration (cells/mL)			
		Mean	SD	Range	*p*-value
HR+HER2−	14 (46.7)	85	123	7–298	0.78
HR-HER2−	10 (33.3)	81	137	5–441	
HR+HER2+	3 (10.0)	36	29	18–70	
HR-HER2+	3 (10.0)	108	180	5–247	

**Table 4.** Cancer treatment received by breast cancer stage.

Treatment type *n* (%)	Number (%) of study participants (*n* = 30)	Stage II (*n* = 4)	Stage III (*n* = 14)	Stage IV (*n* = 12)
Surgery	10 (33.3)	3 (75%)	7 (14%)	0
Chemotherapy	28 (93.3)	3 (750%)	14 (100%)	11 (91.7)
Radiation	5 (16.7)	2 (50%)	3 (21.4%)	0
Palliative care	8 (26.7)	0	1 (7.1)	7 (58.3)

**Table 5.** NK cell concentration and cancer treatment.

Treatment type *n* (%)	Number (%) of study participants (*n* = 30)	NK cell concentration (cells/mL)			
		Mean	SD	Range	*p*-value
Surgery	10 (33.3)	61	44	7–132	0.95
Chemotherapy	28 (93.3)	82	103	5–441	
Radiation	5 (16.7)	53	52	7–132	
Targeted therapy	5 (16.7)	48	40	5–86	
Palliative care	8 (26.7)	96	150	5–441	

## References

[1] Bray F, Ren JS, Masuyer E (2013). Global estimates of cancer prevalence for 27 sites in the adult population in 2008. Int J Cancer.

[2] Bray F, Ferlay J, Soerjomataram I (2018). Global cancer statistics 2018: GLOBOCAN estimates of incidence and mortality worldwide for 36 cancers in 185 countries. CA Cancer J Clin.

[3] Parkin DM, Ferlay J, Jemal A (2018). Cancer in Sub-Saharan Africa.

[4] Pace LE, Shulman LN (2016). Breast cancer in sub-Saharan Africa: challenges and opportunities to reduce mortality. Oncologist.

[5] Espina C, McKenzie F, Dos-Santos-Silva I (2017). Delayed presentation and diagnosis of breast cancer in African women: a systematic review. Ann Epidemiol.

[6] Brinton LA, Figueroa JD, Awuah B (2014). Breast cancer in Sub-Saharan Africa: opportunities for prevention. Breast Cancer Res Treat.

[7] Rahman GA, Olatoke SA, Agodirin SO (2014). Socio-demographic and clinical profile of immune histochemically confirmed breast cancer in a resource limited country. Pan Afr Med J.

[8] Jiagge E, Jibril AS, Chitale D (2016). Comparative analysis of breast cancer phenotypes in African American, white American, and west versus east African patients: correlation between African ancestry and triple- negative breast cancer. Ann Surg Oncol.

[9] Friedl P, Alexander S (2011). Cancer invasion and the microenvironment: plasticity and reciprocity. Cell.

[10] Ferraiuolo RM, Wagner KU (2019). Regulation and new treatment strategies in breast cancer. J Life Sci (Westlake Village).

[11] Vojtek A, Der C (1998). Increasing complexity of the Ras signaling pathway. J Biol Chem.

[12] Toure M, Nguessan E, Bambara AT (2013). Facteurs liés au diagnostic tardif des cancers du sein en Afrique-sub-saharienne: cas de la Côte d'Ivoire [Factors linked to late diagnosis in breast cancer in Sub-Saharan Africa: case of Côte d'Ivoire]. Gynecol Obstet Fertil.

[13] Bates JP, Derakhshandeh R, Jones L (2018). Mechanisms of immune evasion in breast cancer. BMC Cancer.

[14] Ben-Shmuel A, Biber G, Barda-Saad M (2020). Unleashing natural killer cells in the tumor microenvironment-the next generation of immunotherapy?. Front Immunol.

[15] Habif G, Crinier A, André P (2019). Targeting natural killer cells in solid tumors. Cell Mol Immunol.

[16] Ames E, Murphy WJ (2014). Advantages and clinical applications of natural killer cells in cancer immunotherapy. Cancer Immunol Immunother.

[17] Bonilla F, Alvarez-Mon M, Merino F (1990). Natural killer activity in patients with breast cancer. Eur J Gynaecol Oncol.

[18] Bargano RA, Suarez-Alvarez B, Lopez-Larrea C (2014). Secretory pathways generating immunosuppressive NKG2D ligands: new targets for therapeutic intervention. OncoImmunology.

[19] Barsoum IB, Hamilton TK, Li X (2011). Hypoxia induces escape from innate immunity in cancer cells via increased expression of ADAM10: role of nitric oxide. Cancer Res.

[20] Khan M, Arooj S, Wang H (2020). NK cell-based immune checkpoint inhibition. Front Immunol.

[21] Mamessier E, Sylvain A, Thibult M (2011). Human breast cancer cells enhance self-tolerance by promoting evasion from NK cell antitumor immunity. JNCI.

[22] Ascierto ML, Idowu MO, Zhao Y (2013). Molecular signatures mostly associated with NK cells are predictive of relapse free survival in breast cancer patients. J Transl Med.

[23] Peighambarzadeh F, Najafalizadeh A, Esmaeil N (2020). Optimization of *in vitro* expansion and activation of human natural killer cells against breast cancer cell line. Avicenna J Med Biotechnol.

[24] Wambalaba FW, Son B, Wambalaba AE (2019). Prevalence and capacity of cancer diagnostics and treatment: a demand and supply survey of health-care facilities in Kenya. Cancer Control.

[25] Weiner CM, Mathewos A, Addissie A (2018). Characteristics and follow-up of metastatic breast cancer in Ethiopia: a cohort study of 573 women. Breast.

[26] O'Neil DS, Keating NL, Dusengimana JMV (2018). Quality of breast cancer treatment at a rural cancer center in Rwanda. Glob Oncol.

[27] van der Plas WY, Benjamens S, Kruijff S (2020). The increased need for palliative cancer care in Sub-Saharan Africa. Eur J Surg Oncol.

[28] Gayoso I, Sanchez-Correa B, Campos C (2011). Immunosenescence of human natural killer cells. J Innate Immun.

[29] Gounder SS, Abdullah BJJ, Radzuanb NEIBM (2018). Effect of aging on NK cell population and their proliferation at ex vivo culture condition. Anal Cell Pathol (Amst).

[30] Mamessier E, Pradel LC, Thibult ML (2013). Peripheral blood NK cells from breast cancer patients are tumor-induced composite subsets. J Immunol.

[31] Murta EF, Andrade JM, Falcão RP (2000). Lymphocyte subpopulations in patients with advanced breast cancer submitted to neoadjuvant chemotherapy. Tumori.

[32] Fulton A, Heppner G, Roi L (1984). Relationship of natural killer cytotoxicity to clinical and biochemical parameters of primary human breast cancer. Breast Cancer Res Treat.

[33] Muraro E, Martorelli D, Turchet E (2011). A different immunologic profile characterizes patients with HER-2- overexpressing and HER-2-negative locally advanced breast cancer: implications for immune based therapies. Breast Cancer Res.

[34] Verma R, Foster RE, Horgan K (2016). Lymphocyte depletion and repopulation after chemotherapy for primary breast cancer. Breast Cancer Res.

[35] Kim R, Kawai A, Wakisaka M (2020). Immune correlates of the differing pathological and therapeutic effects of neoadjuvant chemotherapy in breast cancer. Eur J Surg Oncol.

[36] Foulds GA, Vadakekolathu J, Abdel-Fatah TMA (2018). Immune-phenotyping and transcriptomic profiling of peripheral blood mononuclear cells from patients with breast cancer: identification of a 3 gene signature which predicts relapse of triple negative breast cancer. Front Immunol.

[37] Larsson AM, Roxå A, Leandersson K (2019). Impact of systemic therapy on circulating leukocyte populations in patients with metastatic breast cancer. Sci Rep.

[38] Sage EK, Schmid TE, Sedelmayr M (2016). Comparative analysis of the effects of radiotherapy versus radiotherapy after adjuvant chemotherapy on the composition of lymphocyte subpopulations in breast cancer patients. Radiother Oncol.

[39] Rothammer A, Sage EK, Werner C (2019). Increased heat shock protein 70 (Hsp70) serum levels and low NK cell counts after radiotherapy - potential markers for predicting breast cancer recurrence?. Radiat Oncol.

[40] The Lancet Oncology (2017). Cancer control in Africa: infrastructure, not philanthropy. Lancet Oncol.

[41] Al-Sukhun S, Lima Lopes G, Gospodarowicz M (2017). Global health initiatives of the International Oncology Community. Am Soc Clin Oncol Educ Book.

[42] Umeh CA, Rockers PC, Laing RO (2020). Pharmaceutical industry-led partnerships focused on addressing the global burden of non-communicable diseases: a review of access accelerated. Public Health.

[43] Gwozdowicz S, Nestorowicz K, Graczyk-Pol E (2019). KIR specificity and avidity of standard and unusual C1, C2, Bw4, Bw6 and A3/11 amino acid motifs at entire HLA:KIR interface between NK and target cells, the functional and evolutionary classification of HLA class I molecules. Int J Immunogenet.

[44] Le Luduec JB, Boudreau JE, Freiberg JC (2019). Novel approach to cell surface discrimination between KIR2DL1 subtypes and KIR2DS1 identifies hierarchies in NK repertoire, education, and tolerance. Front Immunol.

[45] Kennedy PR, Barthen C, Williamson DJ (2019). Genetic diversity affects the nanoscale membrane organization and signalling of natural killer cell receptors. Sci Signal.

